# Phospholipid analysis in sera of horses with allergic dermatitis and in matched healthy controls

**DOI:** 10.1186/s12944-016-0209-4

**Published:** 2016-03-02

**Authors:** Raija Hallamaa, Krishna Batchu

**Affiliations:** Veterinary Clinic, Nummela, Finland; University of Helsinki, Faculty of Veterinary Medicine, Helsinki, Finland; Department of Medical Biochemistry and Developmental Biology, Institute of Biomedicine, University of Helsinki, Helsinki, Finland

**Keywords:** Horse, Allergy, Allergic dermatitis, Lipids, Phospholipids, Insect bite hypersensitivity, Summer eczema, Autoserum therapy

## Abstract

**Background:**

Lipids have become an important target for searching new biomarkers typical of different autoimmune and allergic diseases. The most common allergic dermatitis of the horse is related to stings of insects and is known as insect bite hypersensitivity (IBH) or summer eczema, referring to its recurrence during the summer months. This intense pruritus has certain similarities with atopic dermatitis of humans. The treatment of IBH is difficult and therefore new strategies for therapy are needed. Autoserum therapy based on the use of serum phospholipids has recently been introduced for horses. So far, serum lipids relating to these allergic disorders have been poorly determined. The main aim of this study was to analyse phospholipid profiles in the sera of horses with allergic dermatitis and in their healthy controls and to further assess whether these lipid profiles change according to the clinical status after therapy.

**Methods:**

Sera were collected from 10 horses with allergic dermatitis and from 10 matched healthy controls both before and 4 weeks after the therapy of the affected horses. Eczema horses were treated with an autogenous preparation made from a horse’s own serum and used for oral medication. Samples were analysed for their phospholipid content by liquid chromatography coupled to a triple-quadrupole mass spectrometer (LC-MS). Data of phospholipid concentrations between the groups and over the time were analysed by using the Friedman test. Correlations between the change of concentrations and the clinical status were assessed by Spearman’s rank correlation test.

**Results:**

The major phospholipid classes detected were phosphatidylcholine (PC), sphingomyelin (SM), phosphatidylinositol (PI) and phosphatidylethanolamine (PE). Eczema horses had significantly lower total concentrations of PC (*p* < 0.0001) and SM (*p* = 0.0115) than their healthy controls. After a 4-week therapy, no significant differences were found between the groups. Changes in SM concentrations correlated significantly with alterations in clinical signs (*p* = 0.0047).

**Conclusions:**

Horses with allergic dermatitis have an altered phospholipid profile in their sera as compared with healthy horses and these profiles seem to change according to their clinical status. Sphingomyelin seems to have an active role in the course of equine insect bite hypersensitivity.

## Background

Various new and important insights into the roles of lipids have been demonstrated over the past few decades. Most of these findings are related to inflammatory and immune responses including studies on pro-resolving lipid mediators [1, 2], lipid antigens [3, 4] and lipid presenting CD1 molecules [5–7]. Mass spectrometry with specific software applications has made it possible to identify and quantify a multitude of lipid species both in mammalian cells and also of those existing in circulation [8–12]. Although phospholipids are commonly present in mammalian sera [13, 14], changes in their concentrations under various pathological conditions are so far poorly understood [9, 15, 16]. Presently, there is an increasing need to find new biomarkers feasible for diagnosis, treatment and prognosis – not only in allergic diseases, but also in other immune mediated and neurological disorders [2, 16–23].

**Fig. 1:**
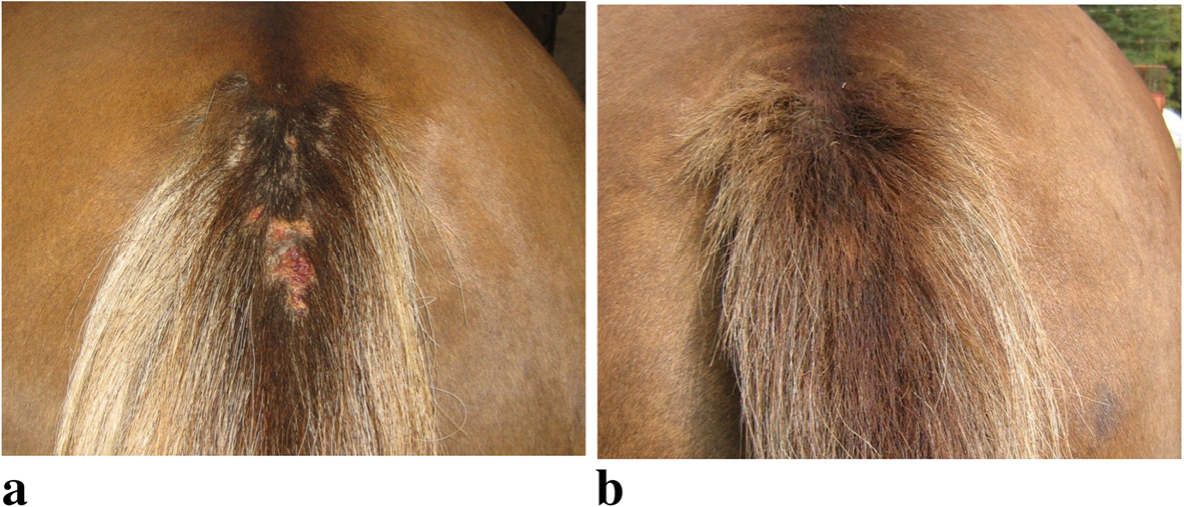
Typical clinical signs in the tail of a horse with insect bite allergic dermatitis (**a**) and the same horse after a 4-week therapy (**b**)

Insect bite hypersensitivity (Fig. 1a) is the most common allergic skin disease of the horse [24] displaying many features similar to atopic dermatitis (AD) of humans [25, 26]. Intense and recurrent pruritus, IgE-mediated responses, perivascular mast cell, eosinophil and T-lymphocyte infiltrations are typical findings of both disorders [25, 27–32]. In addition, horses with insect hypersensitivity show IgE-mediated sensitization also to various environmental allergens [27], as do patients with atopic dermatitis [31, 32]. However, disturbed skin barrier function due to inherited defects of filaggrin has not been studied on horses [26]. Treatment of equine IBH is challenging, since total isolation from biting insects is impossible. Antihistamines or allergen-specific immunotherapy have not been beneficial [33, 34]. Therefore there is a need for comprehensive understanding of this harmful disease. In our previous study [35], we analysed phospholipid contents of autoserum preparations that have been recently used in the therapy of insect hypersensitivity in horses. The idea is to collect remnant lipid particles in these preparations after serial washings [35]. Concentrations of phosphatidylcholine (PC) and sphingomyelin (SM) were significantly more abundant in the preparations made from the sera of the affected than from healthy horses and the amounts of these phospholipids showed significant associations with the severity of prevailing clinical status [35].

The purpose of the current study was to analyse by liquid chromatography-mass spectrometry (LC-MS) phospholipids in the sera of horses with insect bite allergic dermatitis and in their matched healthy controls. We hypothesize that phospholipid profiles may differ between the groups. The additional aim was to assess whether these profiles change according to the clinical status after autoserum therapy.

## Results

The main phospholipid classes analysed were phosphatidylcholine (PC), phosphatidylethanolamine (PE), phosphatidylserine (PS), phosphatidylinositol (PI), phosphatidic acid (PA) and sphingomyelin (SM). Of these, all except for PS were detected across all the samples. Relative contents of the major phospholipid classes before (stage 0) and after the 4-week therapy (stage 4) of eczema horses are listed in Table 1. In addition to the major phospholipid classes, all the samples also contained PA 36:2 and lysoPC species of 16:0, 18:2 and 18:0.

**Table 1:** Relative contents of the major phospholipids detected in sera of the horses

Phospholipids	Relative content (mean ± sd/%)	
	Eczema group	Control group
	Stage 0	
PC	76.0 ± 4.8	83.3 ± 9.8
SM	21.9 ± 3.8	14.8 ± 8.5
PI	1.9 ± 1.4	1.7 ± 1.5
PE	0.2 ± 0.2	0.2 ± 0.2
	Stage 4	
PC	87.4 ± 1.7	85.6 ± 2.7
SM	11.0 ± 1.6	12.4 ± 1.9
PI	1.4 ± 0.7	1.8 ± 1.3
PE	0.2 ± 0.2	0.2 ± 0.2

Samples of horses with allergic dermatitis (*n* = 10) and their matched healthy controls (*n* = 10) are collected before treatment (stage 0) and when affected horses have been on autoserum therapy for 4 weeks (stage 4). Phospholipid classes: phosphatidylcholine (PC), sphingomyelin (SM), phosphatidylinositol (PI), phosphatidylethanolamine (PE).

**Fig. 2:**
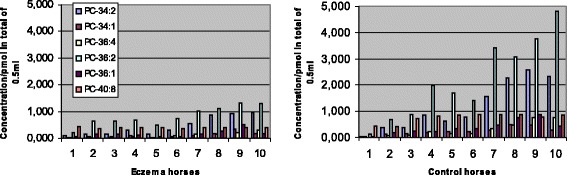
Concentrations of the most abundant phosphatidylcholine (PC) species at the start of therapy. Eczema horses (*n* = 10) and their matched healthy controls (*n* = 10) are numbered similarly. All species showed significantly lower concentrations in eczema horses, at level *P* < 0.0001

**Fig. 3:**
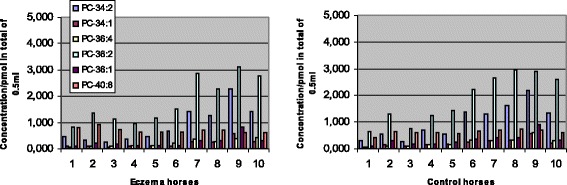
Concentrations of the most abundant phosphatidylcholine (PC) species after a 4-week therapy. Eczema horses (*n* = 10) and their matched healthy controls (*n* = 10) are numbered similarly. Concentrations of PC 34:1 and 36:1 were significantly lower in eczema horses, while 40:8 was in the controls, at level *P* < 0.05

Phosphatidylcholine was the most abundant of all the phospholipid classes detected (Table 1). Its major molecular species in both eczema and control horses at stages 0 and 4 are presented in Figs. 2 and 3, respectively. Of the molecular species, PC 36:2 was the most abundant. Additionally, trace amounts of PC 26:0, 28:0, 31:1, 33:1, 33:2, 33:3, 36:2, 36:3, 36:6 and 38:8 were detected. In the samples collected later in the summer (Table 2), PC concentrations were found to be more abundant than in the samples taken earlier (Figs. 2 and 3). Horses in the eczema group had a significantly lower total concentration of PC than healthy horses before the start of the therapy (Table 3). After the 4-week therapy, no significant difference could be found between the groups (Table 3, Fig. 3). In the eczema group, the total PC concentration had increased significantly, while in the control group there was a small decrease (Figs. 2 and 3). Concentrations of all the major molecular species presented in Figs. 2 and 3 increased significantly (at level *p* < 0.05) in the affected horses between stages 0 and 4, while in the healthy horses, the only significant change observed was a decrease in the concentration of PC 40:8 (*P* = 0.0007).

**Table 2:** Matching of the horses

A	Breed	Gender	Age (years)	Feed	B	Breed	Gender	Age (years)	Feed	Date of 1^st^sample
1	Finnhorse	♀	3	same fodder	1	Finnhorse	♀	9	same fodder	24 April
2	Icelandic horse	♀	22	same fodder	2	Shetland pony	♀	22	same fodder	21 May
3	Icelandic horse	♀	23	same fodder	3	Icelandic horse	♂	9	same fodder	21 May
4	Finnhorse	♂	8	same fodder	4	Finnhorse	♂	7	same fodder	12 June
5	Finnhorse	♂	8	same fodder	5	Finnhorse	♂	7	same fodder	12 June
6	Finnhorse	♀	8	same pasture	6	Finnhorse	♀	8	same pasture	12 June
7	Finnhorse	♀	4	same pasture	7	Finnhorse	♀	7	same pasture	17 July
8	Finnhorse	♀	4	same pasture	8	Finnhorse	♀	6	same pasture	11 Aug.
9	Icelandic horse	♀	10	same fodder	9	Icelandic horse	♀	29	same fodder	7 Sept.
10	Finnhorse	♀	11	same pasture	10	Finnhorse	♀	19	same pasture	12 Sept.

A) 10 horses with allergic dermatitis and B) their 10 matched healthy controls. The horse and its matched control are numbered similarly

**Table 3:** Pairwise comparisons of serum phospholipid concentrations

	PC 0	PC 4	PC 0 c	PC 4 c
PC 0		*p* = 0.0007	*p* < 0.0001	*p* < 0.0001
PC 4	*p* = 0.0007		ns	ns
PC 0 c	*p* < 0.0001	ns		ns
PC 4 c	*p* < 0.0001	ns	ns	
	SM 0	SM 4	SM 0 c	SM 4 c
SM 0		ns	*p* = 0.0115	ns
SM 4	ns		*p* = 0.0005	ns
SM 0 c	*p* = 0.0115	*p* = 0.0005		*p* = 0.0186
SM 4 c	ns	ns	*p* = 0.0186	
	PI 0	PI 4	PI 0 c	PI 4 c
PI 0		ns	ns	ns
PI 4	ns		ns	ns
PI 0 c	ns	ns		ns
PI 4 c	ns	ns	ns	
	PE 0	PE 4	PE 0 c	PE 4 c
PE 0		*p* = 0.0058	ns	*p* < 0.0001
PE 4	*p* = 0.0058		ns	ns
PE 0 c	ns	ns		*p* = 0.0018
PE 4 c	*p* < 0.0001	ns	*p* = 0.0018	

Phosphatidylcholine (PC), sphingomyelin (SM), phosphatidylinositol (PI) and phosphatidylethanolamine (PE) detected from horses with allergic dermatitis and from their matched healthy controls (c); samples collected before (0) and 4 weeks after (4) the therapy of the affected horses

**Fig. 4:**
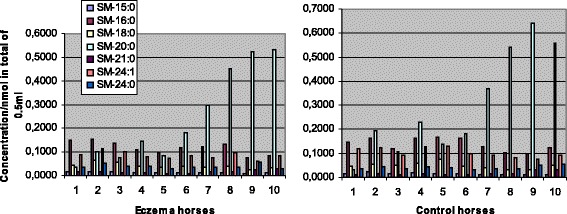
Concentrations of the most abundant sphingomyelin (SM) species at the start of therapy. Eczema horses (*n* = 10) and their matched healthy controls (*n* = 10) are numbered similarly. Species of SM 15:0 and 24:1 showed significantly lower concentrations in eczema horses, at level *P* < 0.05

**Fig. 5:**
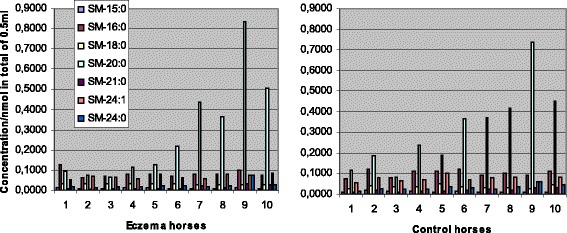
Concentrations of the most abundant sphingomyelin (SM) species after a 4-week therapy. Eczema horses (*n* = 10) and their matched healthy controls (*n* = 10) are numbered similarly. Concentrations of SM 18:0 and 21:0 were significantly lower in eczema horses, at level *P* < 0.05

Concentrations of SM were found to be significantly lower in eczema horses in comparison with their healthy controls at stage 0 (Table 3), although the relative content of SM was higher in the affected horses (Table 1). The total SM concentrations decreased in both groups during the study period (Table 3, Figs. 4 and 5) and at the second sampling there was no significant difference observed between the two groups (Table 3). The major SM species detected and their changes between the stages 0 and 4 are shown in Figs. 4 and 5. The most abundant species was SM 20:0 in both groups and its concentration differed neither between the groups nor the stages. Concentrations of SM 16:0, 18:0, 24:0 and 24:1 decreased significantly (at level *p* <0.05) between the stages in both eczema and healthy horses. Concentrations of SM, like PC, were more abundant in the samples collected later in the summer (Table 2, Figs. 4 and 5).

Phosphatidylinositol and PE displayed small amounts of the phospholipids detected (Table 1). Of PI, molecular species of 32:0, 34:1, 34:2, 36:1, 36:2, 36:3, 36:4 and 38:4 were found. The pairwise comparisons showed no significant differences between the two groups at any stages (Table 3). The detected molecular species of PE were that of 32:0, 36:1, 36:2, 36:3, 36:4, 38:4 and 38:5. Unlike PI, the amounts of PE increased significantly from stage 0 to 4 in both groups (Table 3). However, its relative content did not change due to its small total amount (Table 1). No significant differences between the groups were observed at any of the stages.

**Fig. 6:**
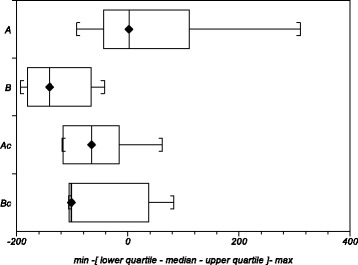
Difference of sphingomyelin (SM) concentrations (pmol in total of 0.5 ml) after therapy. The change between the pre (stage 0-week) and the post (stage 4-week) concentrations in eczema horses with (A, *n* = 6) and without (B, *n* = 4) a positive clinical response and in their matched healthy controls (Ac and Bc, respectively)

Five of the horses had mild and 5 moderate clinical signs at the beginning of the study and the total score of signs was 15, according to the 3-graded scale used. After a 4-week autoserum therapy, the signs had relieved in 6 horses (Fig. 1b), remained unchanged in 3, while one horse suffered from aggravated clinical signs, the total score being 10. Horses with positive clinical outcome showed a milder decrease in SM concentrations than horses with poorer response or controls (Fig. 6). The change in total SM concentrations correlated significantly with alterations in the clinical signs (*P* = 0.0047) and of the major molecular species, SM 15:0 exhibited a significant correlation (*P* = 0.0268). None of the other phospholipid classes showed significant correlations with the change of clinical signs.

No side effects relating to the therapy were observed. Control horses did not develop signs typical of IBH during the study period.

## Discussion

The purpose of this study was to analyse the major phospholipids typical of mammals in the sera of healthy horses and horses with allergic dermatitis. The additional aim was to assess whether profiles of the detected phospholipids change according to clinical status when horses were treated with an autogenous serum preparation. In our previous study [35], we found that autoserum preparations made from the sera of healthy horses and from horses with allergic eczema showed significantly different phospholipid profiles. Additionally, lipid concentrations displayed significant relationship with the severity of clinical signs. Therefore, the present study was focused on serum and changes observed in its lipid profiles in response to autoserum therapy, but not on the clinical efficacy of this therapy *per se*.

Horses with insect hypersensitivity showed significantly lower total concentrations of PC and SM than their matched healthy controls. After a 4-week therapy, those differences faded. The cause of these originally lower concentrations in eczema horses is unclear. Observations from earlier studies indicated that small children with elimination diets or horses fed with oil supplements show altered lipid compositions in their sera [36, 37]. In addition, fasting influences lipid concentrations [9] which stresses the relevance of matching and equal timing when blood is collected from non-fasting horses and their controls. Horses suffering from severe clinical signs may spend time for scratching and thus eat differently than healthy horses. However, none of the horses were so severely affected. Eczema horses were otherwise in a normal physical condition without signs of emaciation and their feeding regimen and daily activities were similar to their controls, neither had the owners observed any changes in their appetite. Although the feeding regimen was similar between the horses, there could be a possibility that some unknown factors might have influenced the lipid concentrations over time, since sera collected in the late summer showed more abundant concentrations of PC and SM than did sera collected earlier in the summer and this trend was detectable in both affected and healthy horses. This emphasizes the importance of matching accompanied by simultaneous samplings in these kinds of studies that are prone to environmental impacts.

Phosphatidylcholine, the major phospholipid present in mammalian sera [13, 14], was found to be the most abundant also in the present study. In humans, alterations in its concentrations have been linked to various pathological conditions, such as cancer, atopic dermatitis and type-1 diabetes, where in altered levels of both PC and SM in sera have been observed [17, 22, 23, 38]. Fuchs et al. showed that in rheumatoid arthritis, PC/lysoPC ratio acted as an indicator of this autoimmune disease and additionally, an increased ratio seemed to be associated with the success of the therapy [39]. LysoPC is a derivative of PC and it is easily formed from PC during storage at room temperature [40]. In horse sera, the relative content of lysoPC is small [14] and therefore it is not an applicable marker in horses as it is in humans. In the present study, lysoPC was detected in minor concentrations and consequently was not evaluated further. Although there was a highly significant difference in PC concentrations between the healthy and the eczema horses at the first sampling, PC did not show any correlation with the change in clinical signs, neither was there any prognostic association between the first PC concentrations and the response to therapy. In contrast to our horse study, higher levels of PC predicted better outcome in patients with atopic eczema after monoclonal antibody therapy [38].

Concentrations of SM were significantly lower in the affected than in healthy horses before the treatment, but not afterwards. Therefore, the elevated relative content of SM in eczema horses at the first sampling is in particular due to the lower levels of PC. Surprisingly, the amounts of SM decreased significantly among the control horses during the study period, which demonstrates that concentrations of certain lipids may fluctuate in healthy horses, at least to some extent without largely undermining the relative lipid content. This fluctuation possibly resulted from environmental changes, since SM concentrations decreased also in eczema horses. However, the decrease was evident only in those eczema horses that showed a poorer outcome in response to therapy. This suggests that affected horses have a specific need for SM during recovery. Sphingomyelins are important lipids involved in multiple biochemical reactions, especially in the skin and nervous tissue [15, 41–43]. These phospholipids are necessary not only as structural components, but also as bioactive regulators and messengers in cutaneous homeostasis and immune mediated responses, particularly via the main derivative, sphingosine-1-phosphate [41, 42, 44, 45]. Sphingosine-1-phosphate (S1P) plays a crucial part in mast cell regulation by affecting mast cell degranulation and by activating other immune cells involved [44, 45]. The engagement of S1P to its receptors on the plasma membrane seems to be the most critical point in the pathway of mast cell activation [44–46]. In addition to S1P, ceramide is another important metabolite of SM and is one of the main lipid components in skin barrier [41]. In the affected skin, ceramides are continuously utilised for repairing [41]. Although various lipid combinations and their changes in response to therapy have been linked to atopic dermatitis in humans [38, 47], SM seems to be a potential player in insect bite allergy of the horse, since alterations in its concentrations were significantly associated with changes in clinical signs. Similarly, sphingolipids have been shown to act as biomarkers in delayed type hypersensitivity of mice [16].

In our study, both PI and PE were detected in minor amounts, which is consistent with the previous studies [14]. These phospholipids are probably not involved in insect bite hypersensitivity, since there was no difference between the groups in the first sampling and the concentrations of PE changed similarly in both groups. Traces of PA were found in all samples and as an inner leaflet phospholipid, it is not normally detected from the horse serum [14]. Presence of PA is probably related to blood cells that remained when the samples were harvested without centrifugation [35].

Horses in the present study were treated with an autoserum preparation and phospholipids were analysed in response to this treatment. However, we had no control group for the therapy; therefore it is not relevant to conclude that the observed changes could be specific for the current treatment. In fact, further studies are needed to evaluate a relationship between phospholipid concentrations and responses to therapy, comprising placebo or other therapeutic interventions and a greater number of horses than those enrolled in this study.

The lower concentration of PC and SM detected in the sera of affected horses was an unexpected finding, since our earlier study demonstrated that autoserum preparations made from the sera of horses with allergic dermatitis showed significantly higher concentrations of PC and SM than the preparations made from the sera of healthy horses [35]. This contradictory result may be associated with the hydrophobic/hydrophilic interactions of the lipid particles. The serum for autoserum preparations was collected from the superficial layer of the blood sample, washed twice with water and finally added in ethanol; all consecutive transfers were collected from the superficial layer of the previous solution [35]. Thus the most hydrophobic and water-insoluble lipid molecules were presumably concentrated in such autoserum preparations. Instead of washings, samples of the present work were mixed directly with alcohol. Therefore, these samples included not only the most hydrophobic but also the less hydrophobic lipid particles, such as PC 26:0 with a short acyl chain or PC 36:6, 38:8 and 40:8 with several double bonds; species that were not detected in autoserum preparations [35]. The discrepancy with the amounts of PC and SM in the serum *versus* autoserum preparations may be interpreted as so far unknown, hydrophilic/hydrophobic interactions possibly linked to mineral salts that have been found to affect peculiarly the distribution of lipids after successive washings [48]. Sera of affected horses may contain those mineral salts that make lipids less water-soluble and enable them to concentrate into autoserum preparations, although according to the current study the total concentrations of PC and SM were lower in these horses. This interpretation could explain with caution the underlying mechanism of this therapy, possibly culminating in the ability of signalling molecules to engage their corresponding receptors which has been recognized to be a critical event for the following mast cell responses [44–46].

## Conclusions

Horses with an allergic dermatitis have an altered lipid profile in their sera as compared with healthy controls and these differences seem to change according to the clinical status of the horses. Sphingomyelin shows a functional role in equine insect bite hypersensitivity. Lipid profiling may become an important target for further studies, since relevant fingerprints delineating different pathological disorders are needed – in both human and veterinary medicine.

## Methods

### Horses

A total of 20 horses entered in this study in 2014, between the seasons of spring and autumn, the period when horses are exposed to bites of insects. The horses comprised 10 animals with clinical signs of insect bite hypersensitivity (eczema group) and 10 matched healthy controls (control group). The matching is presented in Table 2. Clinical signs of eczema were categorised as mild, moderate or severe at the time of blood sampling and scored 1–3, respectively. Signs were mild, if the horse had only pruritus without skin lesions. Pruritus with mild skin affections in the mane, tail (Fig. 1a) and/or body was graded as moderate, while pruritus over large skin lesions was regarded as a severe sign. Clinical signs were evaluated after the 4-week therapy and considered as relieved when signs became milder on this scale (Fig. 1a and b). The changes in clinical signs were recorded as relieved, no change or aggravated and respectively scored as −1, 0 or +1. Five of the horses had mild and 5 moderate clinical signs in the beginning of the study. The matched healthy controls without a history of IBH lived in the same farms and were fed with the similar fodder as their affected counterparts. Horses were also matched with breed, gender and age as closely as possible (Table 2). The eczema horse and its matched control are numbered similarly in the tables and figures throughout this article. This study has been approved by the Regional State Administrative Agency of Southern Finland (ESAVI/1016/04.10.07/2014) and owners’ signed permission for inclusion was obtained.

### Blood sampling

The first sample (marked as stage 0 in text, tables and figures) was taken when the horse had shown typical clinical signs of IBH for at least 2 weeks. Blood sample was first collected using 10 ml plain vacuum tubes that were filled in full and kept at room temperature for at least 3 hours before harvesting. The serum was harvested without using a centrifuge, thus avoiding destruction of serum lipids. With a calibrated pipette, 0.065 ml of the serum was taken from the superficial layer and stored in 4.2 ml of 48 % ethanol. The second sample (marked as stage 4 in text, tables and figures) was collected after a 4-week therapy of the affected horse and handled equally. Samples were drawn from the counterpart horse similarly and simultaneously.

### Therapy of the eczema horses

All horses in the eczema group were treated with an oral administration of an autoserum preparation for 4 weeks. Sera used for the preparations were drawn at the time of the first blood sampling. Sera were harvested without using a centrifuge and 0.065 ml of the serum was taken from the superficial, lipid-containing layer of the tube. Lipid particles were washed twice with sterile water (1:100), accordingly 0.065 ml collected from the superficial layer of the previous dilution. After the second washing, 0.065 ml was taken from the superficial layer of the latter solution and added finally in 48 % ethanol (1:100) and absorbed in sugar granules [49]. A dose of granules was given orally once a day for 2 weeks, followed by a 1-week pause, after which horses were medicated for one further week. Horses in the control group received no therapy.

### Lipid analysis of the sera

Phospholipid classes analysed were PC, PE, PS, PI, PA and SM. All the samples stored in ethanol were subjected to Folch’s method [48] for lipid extraction, dried under a N_2_ stream, reconstituted in 500 μl chloroform/methanol (1:2) and further spiked with the following labelled standards^a^ corresponding to each head group: D_9_(di-44:2) and D_9_(di-40:2) for PC, D_4_(di-40:2) and D_4_(di-20:0) for PE, D_3_(di-40:2) and D_3_(di-44:2) for PS, D_6_(di-36:2) and D_6_(di-28:0) for PI and finally unlabelled 25:0-SM. Liquid chromatography-mass spectrometry (LC-MS) with selective reaction monitoring (SRM) was used for the analyses. Waters ACQUITY Ultra Performance LC system^b^ equipped with a Waters ACQUITY BEH C_18_ column (1.0 × 100 mm) was used to separate the molecular species using gradient elution. Solvent A was acetonitrile/H_2_O (60:40) with 10 mM ammonium formate and 1 % NH_4_OH, while solvent B was isopropanol/acetonitrile (90:10) containing 10 mM ammonium formate and 1 % NH_4_OH. The flow rate was 0.13 ml/min and the column temperature 60 °C. Solvent B was set to 40 % at injection and increased linearly to 100 % in 14 min, remained at this value for 3 min, decreased back to 40 % in 1 min and then remained there until the end of the gradient at 20 min. The eluent was directed to the electrospray ionization (ESI) source of Waters Quattro Premier triple-quadrupole mass spectrometer^b^ operated in the positive ion mode. For SRM transitions, proton adducts of the PC, PE, PS and PI species were selected as the precursors, while the product ion was either the head group (PC, SM, PI) or the diacylglycerol fragment (PE, PS). For quantification purposes, the SRM chromatograms were integrated and the relative concentrations of the individual molecular species were calculated using QuanLynx software^b^.

Researchers processing the samples for mass spectrometric analyses were not aware of the horses’ grouping. The researcher who performed clinical examinations recorded these results to another, independent researcher before the lipid analyses were made. When all studies were completed, the clinical results were combined to corresponding lipid profiles under the control of the independent supervisor.

### Data analysis

The Shapiro-Wilk test was used to assess the normality of lipid concentration data. Uniformly parametric distribution was found neither in the separate phospholipids classes nor at the different stages. Therefore, pairwise comparisons of phospholipid concentrations between the matched groups and over the time were analysed by using the Friedman test. The relationship between the change of phospholipid concentrations and clinical signs was evaluated by Spearman’s correlation test. Analyses were performed using statistical software StatsDirect^c^. Two-sided P values <0.05 were considered significant.
